# Clinical characteristics and diagnostic challenges of non-ST-segment elevation acute coronary syndrome patients with normal electrocardiograms: a review

**DOI:** 10.3389/fcvm.2026.1835982

**Published:** 2026-08-06

**Authors:** Yiwen Zhang, Youlu Shen

**Affiliations:** Department of Cardiology, Affiliated Hospital of Qinghai University, Xining, China

**Keywords:** clinical characteristics, diagnostic algorithm, diagnostic challenges, high-sensitivity cardiac troponin, multimodality imaging, non-ST-segment elevation acute coronary syndrome, normal electrocardiogram, prognosis

## Abstract

Non-ST-segment elevation acute coronary syndrome (NSTE-ACS) represents a major subtype of acute coronary syndrome (ACS). A clinically relevant but often overlooked subgroup of patients presents with typical ischemic symptoms despite a normal initial 12-lead electrocardiogram (ECG), creating real diagnostic difficulty. This narrative review synthesizes current evidence across several domains: epidemiology, pathophysiology, clinical presentation, diagnostic evaluation, and management of NSTE-ACS patients with normal ECGs. A structured literature search of PubMed, Web of Science, and the China National Knowledge Infrastructure (CNKI) databases, with emphasis on prospective cohort studies, registries, systematic reviews, meta-analyses, and current European Society of Cardiology (ESC) and American College of Cardiology/American Heart Association (ACC/AHA) guideline recommendations. Depending on the definition of “normal ECG” and study population, approximately 1%–8% of confirmed NSTE-ACS patients present with a normal initial ECG. These patients frequently exhibit single-vessel disease, non-obstructive coronary artery disease, or alternative mechanisms such as coronary vasospasm and microvascular dysfunction. High-sensitivity cardiac troponin (hs-cTn) assays using rapid 0/1-h or 0/2-h algorithms constitute the diagnostic cornerstone, while coronary computed tomography angiography (CCTA) provides valuable anatomical assessment, particularly in low-to-intermediate-risk patients. This review presents an integrated diagnostic algorithm, critically evaluates the complementary roles of multimodality imaging techniques, and discusses future directions including artificial intelligence-assisted ECG interpretation and wearable monitoring technologies.

## 1 Introduction

Non-ST-segment elevation acute coronary syndrome (NSTE-ACS), encompassing unstable angina (UA) and non-ST-segment elevation myocardial infarction (NSTEMI), is a major clinical entity caused by myocardial ischemia resulting from partial or intermittent coronary artery occlusion, without persistent ST-segment elevation on the surface electrocardiogram (ECG) (1). The initial evaluation of NSTE-ACS relies primarily on clinical presentation, ECG changes, and cardiac biomarkers—particularly high-sensitivity cardiac troponin (hs-cTn) (2). However, a clinically important subgroup of patients presents with typical ischemic chest pain despite a normal 12-lead ECG at the time of initial assessment. This scenario creates a significant diagnostic dilemma, as the absence of ECG abnormalities may lead to underestimation of risk, delayed diagnosis, and adverse outcomes (3, 4).

The prevalence of this phenomenon is not negligible. The numbers are telling. Depending on the study population and the definition of “normal ECG,” approximately 1%–8% of confirmed NSTE-ACS patients present with a normal initial ECG (1–3). Interestingly, among ACS patients who initially present with a normal ECG, approximately 23%–27% are subsequently diagnosed with NSTEMI based on elevated troponin levels (1, 2). These two metrics reflect different perspectives: the former quantifies the proportion of NSTE-ACS patients with normal ECG, while the latter captures the proportion of normal-ECG ACS patients who ultimately prove to have NSTEMI. Taken together, these data call into question the reliability of ECG alone for excluding myocardial ischemia and point to the need for a multimodal diagnostic approach.

The pathophysiological mechanisms underlying a normal ECG in NSTE-ACS involve multiple factors, including the extent, location, and duration of ischemia, as well as the presence of collateral circulation. Subendocardial or limited ischemia may not generate sufficient electrical changes for detection by standard 12-lead ECG, while transient ischemic episodes may resolve before ECG recording (5, 6). Additionally, non-atherosclerotic causes, including coronary vasospasm, microvascular dysfunction, and myocardial bridging, may produce ACS-like symptoms with minimal or no ECG changes (7, 8).

Advances in diagnostic technology have improved the detection and risk stratification of these patients. High-sensitivity troponin assays enable earlier and more accurate identification of myocardial injury even without ECG changes (9). Coronary computed tomography angiography (CCTA) and stress echocardiography provide useful anatomical and functional information (10–12). Emerging ECG analysis techniques, including synthesized 18-lead ECG and machine learning-enhanced algorithms, show promise in detecting subtle ischemic changes (13, 14). Despite these advances, management is still difficult, and clear guidance for this specific subgroup within current guidelines is limited.

This review aims to systematically synthesize the clinical characteristics, diagnostic challenges, and evolving management strategies for NSTE-ACS patients presenting with normal ECGs, with particular emphasis on integrating current guideline recommendations, presenting an evidence-based diagnostic algorithm, and identifying future research directions. By critically evaluating the available evidence and identifying knowledge gaps, this review seeks to improve clinical awareness and optimize patient outcomes.

## 2 Methods

A comprehensive literature search was conducted across PubMed, Web of Science, and the China National Knowledge Infrastructure (CNKI) databases, covering the period from January 2000 to March 2026. The search strategy employed a combination of Medical Subject Headings (MeSH) terms and free-text keywords related to non-ST-segment elevation acute coronary syndrome (NSTE-ACS), normal electrocardiogram, diagnosis, biomarkers, imaging, risk stratification, and prognosis. Particular emphasis was placed on prospective cohort studies, registries, systematic reviews, meta-analyses, and current European Society of Cardiology (ESC) and American College of Cardiology/American Heart Association (ACC/AHA) guidelines (48–53).

## 3 NSTE-ACS with normal ECG: epidemiology and definition challenges

### 3.1 Incidence and population characteristics

Patients with NSTE-ACS who present with a completely normal 12-lead ECG at initial evaluation constitute a clinically challenging subgroup. Reported prevalence varies considerably across studies, ranging from approximately 1%–8% of confirmed NSTE-ACS cases, depending on the study population, the definition of “normal ECG,” and whether serial ECGs were obtained (1–3). In the cross-sectional studies by Khan et al. (1) and Zeb et al. (2) from Pakistani cardiac centers, approximately 22.8%–26.7% of ACS patients with normal ECGs were subsequently diagnosed with NSTEMI based on elevated troponin. While these studies provide valuable prevalence data from South Asian populations, their single-center design and potential selection bias limit generalizability. The larger retrospective study by Ljubojevic et al. (3) involving over 3,000 patients found a lower rate of 4.1%, likely reflecting stricter ECG criteria and a different patient demographic. This discrepancy highlights the methodological challenge of defining “normal ECG” across studies.

What defines “normal” matters. Clinically, patients in this subgroup tend to be younger, with less prior myocardial infarction history, and a higher proportion of female patients, consistent with the higher prevalence of microvascular angina and atypical symptom presentation in women (3, 15). We believe a critical observation is that some patients with normal ECGs harbor severe coronary artery lesions, including complete occlusion, which are not reflected on surface ECG (16, 17). This highlights the heterogeneity of this population—and the danger of equating a normal ECG with benign pathology. The study by Bruno et al. (17), a real-world prospective cohort, demonstrated that occlusion of the infarct-related artery can present without ST-elevation, emphasizing that the ECG is an insensitive marker for coronary occlusion in certain anatomical configurations.

### 3.2 Definition and evolution of “normal ECG”

The definition of “normal ECG” in the context of suspected NSTE-ACS is nuanced. It requires exclusion of any non-specific ST-T changes, prior Q waves, conduction abnormalities, or repolarization variants that may interfere with interpretation (18, 19). This demands expertise in ECG interpretation, as subtle abnormalities may be overlooked. A key consideration is that the ECG is a dynamic tool; ischemic changes may be transient, occurring only during chest pain episodes and resolving during asymptomatic periods (19). Continuous or serial ECG monitoring can detect intermittent ST-segment changes that a single 12-lead recording would miss. The addition of posterior wall leads (V7–V9) can reveal posterior wall ischemia not apparent on standard leads (13, 19).

Advanced ECG technologies, including high-resolution digital ECG and computer-aided analysis, may help detect subtle repolarization abnormalities (20). Quantitative parameters such as the T-wave/QRS ratio and ST-segment integral have been investigated as supplementary diagnostic tools, with studies showing correlations with ischemia severity (21, 22). That said, these technologies and parameters remain primarily investigational, and their clinical applicability is limited by the lack of large-scale validation studies—a gap that remains to be filled. The ongoing refinement of ECG analysis techniques highlights the importance of integrating clinical context, serial ECGs, biomarker trends, and imaging to refine the definition of a truly “normal” ECG and improve diagnostic accuracy.

## 4 Pathophysiological basis and coronary artery lesion characteristics

### 4.1 Ischemic mechanisms and causes of ECG silence

Several pathophysiological factors contribute to the absence of typical ischemic ECG changes in NSTE-ACS. First, the spatial extent of ischemia plays a critical role. Subendocardial or limited ischemia may not produce sufficient electrical activity changes detectable by standard 12-lead ECG, which predominantly reflects transmural electrical activity (18). Second, the anatomical location matters: ischemia in territories supplied by the left circumflex artery (e.g., posterior or lateral wall) is frequently underrepresented on standard leads, leading to underestimation of ischemic changes (3, 23). Third, transient ischemia may resolve before ECG recording, normalizing the waveform (19). Fourth, well-developed collateral circulation may preserve myocardial perfusion and attenuate ECG changes (16).

Together, these factors explain why a substantial proportion of NSTE-ACS patients, including those with confirmed NSTEMI, may present without obvious ECG abnormalities. However, it should be acknowledged that most evidence supporting these mechanistic explanations derives from small observational studies or post hoc analyses; large-scale prospective studies systematically correlating intracoronary imaging findings with surface ECG patterns in well-characterized normal-ECG NSTE-ACS cohorts remain lacking. Clinically, this means that triage decisions should not rest on a single ECG recording, and a broader approach incorporating biomarkers and imaging is essential. The finding that continuous ST-segment monitoring can detect transient ischemic episodes that would otherwise be missed further supports the value of prolonged ECG surveillance in selected patients (19, 24).

### 4.2 Coronary anatomy and plaque characteristics

Coronary angiography studies reveal that NSTE-ACS patients with normal ECGs often exhibit distinct coronary involvement patterns compared to those with typical ECG changes. Culprit lesions are frequently located in non-left anterior descending (LAD) arteries, such as the left circumflex or right coronary artery, or present as single-vessel rather than multivessel disease (3, 25). The culprit plaques are often lipid-rich vulnerable plaques whose rupture produces non-occlusive thrombus, resulting in intermittent rather than persistent occlusion—a mechanism that may explain why the ECG looks deceptively normal (25).

Recognizing the value of plaque characterization, intravascular imaging using optical coherence tomography (OCT) has further elucidated that some patients exhibit plaque erosion rather than rupture, characterized by smaller thrombus burden and less distal embolization, potentially leading to more occult ischemia (25). Yet most evidence on plaque morphology in this specific subgroup comes from small observational studies and *post hoc* analyses, and large prospective studies specifically examining NSTE-ACS patients with normal ECG are lacking. Myocardial bridging, a congenital anomaly causing dynamic coronary compression during systole, has been described as a rare cause of ischemia with normal ECG (4), but the quality of this evidence is limited to isolated case reports, and its true prevalence and clinical significance in this population remain uncertain. These gaps point to the need for prospective studies employing intravascular imaging in well-characterized cohorts of NSTE-ACS patients with normal ECGs.

## 5 Clinical presentation, biomarkers, and risk assessment

### 5.1 Symptomatology and differential diagnosis

The clinical presentation of NSTE-ACS with normal ECG is complicated by symptom overlap with non-cardiac conditions. While chest pain may retain characteristics of typical angina—substernal location, exertional trigger, and radiation to the left arm or jaw—the absence of ECG abnormalities frequently leads to confusion with gastroesophageal reflux disease, musculoskeletal pain, or anxiety-related chest discomfort. Studies from Pakistani cardiac centers demonstrated that a considerable proportion of NSTE-ACS patients had normal ECGs at presentation, with diagnosis confirmed only after biomarker testing (1, 2). The data are striking.

It reinforces the need to combine clinical assessment with serial biomarker measurement.

Accompanying symptoms such as dyspnea, diaphoresis, and nausea, while common, lack specificity in the absence of ECG support. Rare causes of ACS-like presentations—including left atrial myxoma, coronary vasospasm, pheochromocytoma-induced myocardial injury, and leukemia-associated coronary thrombosis (6, 7, 26)—have been described in case reports. While these reports are educationally valuable, they represent exceptional circumstances and should not be overemphasized relative to the far more common atherosclerotic mechanisms. Even when the overall ECG appears normal, subtle patterns such as the Aslanger sign may indicate significant coronary artery stenosis (27). Clinicians must maintain a high index of suspicion and integrate clinical, biochemical, and imaging data to distinguish true ischemic chest pain from mimics.

### 5.2 High-sensitivity cardiac troponin: the diagnostic cornerstone

The introduction of high-sensitivity cardiac troponin (hs-cTn) assays has revolutionized diagnosis of NSTE-ACS, particularly in patients with normal or ambiguous ECGs. Even a slight hs-cTn elevation or dynamic change can identify myocardial injury consistent with NSTEMI, overcoming the limitations of ECG-based diagnosis (1, 2, 55). The ESC 2020 NSTEMI-focused update (48) and the 2023 ESC ACS guidelines (49) strongly endorse rapid hs-cTn algorithms (0/1-h or 0/2-h protocols) for early rule-in and rule-out of NSTEMI, based on extensive validation in large multicenter cohorts (9, 56, 57).

The study by Feng et al. (9) specifically evaluated the performance of the ESC 0/1-h hs-cTnI algorithm in suspected NSTEMI patients with normal vs. abnormal ECGs, providing direct evidence that rapid algorithms maintain diagnostic accuracy in the normal-ECG subgroup. This is a methodologically rigorous study that addresses a critical evidence gap. However, the generalizability may be limited by the single-center design and the specific hs-cTnI assay used. The magnitude of troponin elevation correlates with infarct size and provides prognostic information; higher hs-cTn levels are associated with increased risk of major adverse cardiovascular events (MACE) (28).

A critical limitation of hs-cTn is its lack of specificity for ischemic injury. Elevated troponin can be observed in numerous non-ischemic conditions, including myocarditis, Takotsubo cardiomyopathy, sepsis, renal failure, and pulmonary embolism (29, 30). Therefore, troponin elevation must always be interpreted in clinical context, and dynamic changes (rise and/or fall) are more indicative of acute injury than single measurements. The combined use of hs-cTn with other biomarkers, such as B-type natriuretic peptide (BNP) and growth differentiation factor-15 (GDF-15), has been explored to improve diagnostic and prognostic accuracy (28), though their incremental value in the specific normal-ECG subgroup has yet to be clarified. Table 1 provides a comparative summary of key cardiac biomarkers used in NSTE-ACS diagnosis.

**Table 1:** Comparison of Key cardiac biomarkers in NSTE-ACS diagnosis.

Biomarker	Diagnostic role	Advantages	Limitations	Guideline status
hs-cTnT/hs-cTnI	Primary diagnostic marker for myocardial injury	High sensitivity; enables early detection (0/1 h algorithms); quantifies infarct size; strong prognostic value	Non-specific for ischemia (elevated in myocarditis, sepsis, renal failure); requires dynamic measurement; assay-dependent cutoffs	ESC Class I, Level A; ACC/AHA Class I, Level A
CK-MB	Secondary marker; detects reinfarction	Useful for detecting early reinfarction; widely available	Less sensitive than hs-cTn; replaced by hs-cTn in most algorithms; limited added value	ESC Class IIa; ACC/AHA Class IIb
BNP/NT-proBNP	Prognostic marker; risk stratification	Predicts mortality and heart failure; complementary to troponin	Not diagnostic for ACS; elevated in heart failure and other conditions	ESC Class IIb for risk stratification
GDF-15	Prognostic biomarker	Independent predictor of MACE and mortality; identifies high-risk patients	Limited clinical availability; not universally validated; investigational	ESC Class IIb; investigational
hs-CRP	Inflammatory risk marker	Identifies residual inflammatory risk; may guide anti-inflammatory therapy	Non-specific; limited diagnostic value for acute ACS	Not recommended for routine ACS diagnosis
D-dimer	Exclusion of thromboembolic disease	Useful for differential diagnosis (PE, aortic dissection)	Non-specific; elevated in many conditions; not diagnostic for ACS	Adjunct for differential diagnosis
Copeptin	Early rule-out adjunct with hs-cTn	Rises rapidly; combined with troponin improves early rule-out	Limited data in normal-ECG subgroup; not widely available	ESC Class IIb for early rule-out

### 5.3 Applicability of risk scoring tools

Traditional risk scoring systems present both opportunities and challenges in NSTE-ACS patients with normal ECG. The Global Registry of Acute Coronary Events (GRACE) score and the Thrombolysis in Myocardial Infarction (TIMI) risk score have been validated in broad ACS populations but incorporate ECG abnormalities as key variables, potentially underestimating risk in patients without ECG changes (3, 9). The ESC guidelines recommend the GRACE score for risk stratification (Class I, Level A), but acknowledge its limitations in specific subgroups. The HEART score, which balances history, ECG, age, risk factors, and troponin, has shown promise in this context because it assigns proportional weight to clinical history and biomarkers, partially compensating for the absence of ECG changes (16). That said, the HEART score has been primarily validated in emergency department chest pain populations, and its specific performance in confirmed NSTE-ACS with normal ECG requires further study. This matters clinically.

The 2021 ACC/AHA Chest Pain Evaluation guidelines recommend the use of the HEART pathway for patients with suspected ACS, with specific thresholds for low-risk (HEART ≤ 3) and high-risk (≥7) stratification. The ESC 2023 ACS guidelines further emphasize the integration of hs-cTn algorithms with clinical risk scores. Emerging biomarkers such as GDF-15 and BNP are being investigated for their incremental prognostic value (31), while advanced imaging methods, including speckle-tracking echocardiography for global longitudinal strain assessment, provide complementary risk information (11, 32). Machine learning approaches integrating multidimensional clinical, biomarker, and imaging data offer a potentially useful avenue for personalized risk assessment (33), though these are still investigational and need prospective validation.

## 6 Imaging and functional assessment strategies

### 6.1 Noninvasive imaging modalities: complementary roles

For patients with suspected NSTE-ACS and normal ECG, noninvasive imaging is central to diagnosis, risk stratification, and treatment decisions. The selection of imaging modality should be guided by patient risk profile, hemodynamic stability, renal function, and the specific clinical question being addressed. Each modality has distinct advantages and limitations (summarized in Table 2).

**Table 2:** Comparison of imaging modalities in NSTE-ACS with normal ECG.

Modality	Primary role	Key advantages	Key limitations	Best suited for
CCTA	Anatomical exclusion of CAD	High negative predictive value; fast; non-invasive; identifies plaque characteristics	Limited functional data; artifacts with calcification/stents; radiation; requires HR control	Low-to-intermediate risk patients; rule-out CAD
CMR	Tissue characterization; differential diagnosis	Detects edema, fibrosis, microinfarction; no radiation; differentiates ACS mimics	Limited availability; long scan time; contraindicated with certain devices; unstable patients	Inconclusive initial workup; suspected myocarditis/Takotsubo
Stress Echo (with GLS)	Functional ischemia detection	Widely available; real-time; no radiation; GLS quantification	Operator-dependent; acoustic window limitations; lower sensitivity for single-vessel disease	Intermediate risk; suspected microvascular dysfunction
MPI (SPECT/PET)	Perfusion defect assessment	Prognostic value; anatomical + functional; risk stratification	Radiation (SPECT); limited spatial resolution; balanced ischemia false negatives; limited availability (PET)	Intermediate-to-high risk; multi-vessel assessment
IVUS	Intracoronary plaque assessment	Cross-sectional wall imaging; plaque burden; calcium; remodeling	Invasive; cost; additional procedural time; lower resolution than OCT	PCI optimization; angiographically ambiguous lesions
OCT	High-resolution plaque characterization	Highest resolution; identifies TCFA, rupture, erosion, thrombus	Invasive; limited penetration depth (∼1–2 mm); requires contrast flush	Diagnosing plaque erosion vs. rupture; stent optimization
FFR/iFR	Functional significance of stenosis	Guides revascularization decisions; improves outcomes; evidence-based	Invasive (FFR); vasodilator required (FFR); wire-based	Intermediate angiographic stenosis (40%–70%)

#### 6.1.1 Coronary computed tomography angiography (CCTA)

CCTA has emerged as a first-line noninvasive anatomical imaging modality, particularly valuable for low- to intermediate-risk patients with normal ECG and negative or borderline troponin. CCTA directly visualizes coronary anatomy, enabling exclusion of obstructive coronary artery disease (CAD) as well as identification of non-obstructive plaques that functional testing alone might miss. A systematic review and meta-analysis by Tateishi et al. (34) showed that CCTA in the emergency department setting can safely exclude ACS, shorten hospitalization time, and reduce medical costs without increasing adverse events. The ongoing TRACTION trial (35), a randomized comparison of CCTA vs. invasive coronary angiography for interventional triage in ACS, will provide important evidence on the optimal role of CCTA. The COURSE trial (10) is specifically investigating CCTA for patients with acute chest pain and low-range positive hs-cTn—a population highly relevant to the normal-ECG NSTE-ACS subgroup. CCTA does have limitations, though: it provides limited functional information, requires adequate heart rate control, and its diagnostic accuracy is reduced in patients with heavy calcification or prior stents. Moreover, most evidence supporting CCTA in the acute chest pain pathway comes from studies conducted in high-resource settings; its generalizability to centers without ready access to advanced imaging remains uncertain.

#### 6.1.2 Cardiac magnetic resonance imaging (CMR)

By providing unique tissue characterization capabilities, CMR can detect myocardial edema, fibrosis, and microinfarction through late gadolinium enhancement (LGE) and T1/T2 mapping sequences. This is particularly valuable for differentiating true ischemic injury from ACS mimics such as myocarditis or Takotsubo cardiomyopathy (36). CMR can identify subendocardial infarction patterns that may not be detected by ECG or echocardiography. CMR, on the other hand, has practical limitations: limited availability, longer acquisition times, contraindication in patients with certain implanted devices, and difficulty in monitoring hemodynamically unstable patients. Its role is primarily as a problem-solving tool when initial evaluation is inconclusive rather than as a first-line modality in the acute setting.

#### 6.1.3 Stress echocardiography

Stress echocardiography, including exercise and pharmacological (dobutamine or vasodilator) protocols, detects inducible wall motion abnormalities caused by ischemia. Speckle-tracking echocardiography (STE) enables quantitative assessment of global longitudinal strain (GLS), which has been shown to identify significant coronary artery disease in patients with suspected unstable angina (11) and to predict the culprit vessel in NSTE-ACS (12). The study by Karlsen et al. (11) demonstrated that exercise-induced reduction in GLS can rule out significant CAD, providing a functional complement to anatomical imaging. Stress echocardiography is widely available, radiation-free, and allows real-time assessment, but its accuracy is operator-dependent and limited by inadequate acoustic windows in some patients.

#### 6.1.4 Myocardial perfusion imaging (MPI)

Single-photon emission computed tomography (SPECT) myocardial perfusion imaging evaluates perfusion defects during stress and rest, providing both anatomical and functional information. The study by Kraen et al. (37) demonstrated the incremental prognostic value of exercise ECG combined with MPS for predicting cardiac events. MPI is particularly useful for risk stratification in patients with intermediate pretest probability. That said, radiation exposure, limited spatial resolution (especially for small subendocardial defects), and the potential for false-positive results in patients with multi-vessel balanced ischemia are important limitations. Positron emission tomography (PET) perfusion imaging offers superior spatial resolution and quantitative blood flow assessment but is less widely available.

#### 6.1.5 Intravascular ultrasound (IVUS) and optical coherence tomography (OCT)

Used during invasive coronary angiography, IVUS and OCT evaluate plaque morphology in detail as intracoronary imaging modalities. IVUS provides cross-sectional images of the coronary artery wall, allowing assessment of plaque burden, remodeling, and calcium distribution. OCT offers higher resolution imaging, allowing identification of thin-cap fibroatheromas, plaque rupture, and erosion (25). Both modalities can detect lesions that may be missed by angiography alone, including angiographically occult plaques and dissections. Their use is recommended by ESC guidelines (Class IIa) for optimizing percutaneous coronary intervention and for diagnosing non-obstructive coronary pathology. However, they are invasive, add cost and procedural time, and are not available in all catheterization laboratories.

#### 6.1.6 Physiological assessment: fractional flow reserve (FFR) and non-invasive FFR

FFR, measured during invasive coronary angiography using a pressure wire, assesses the functional significance of intermediate coronary stenoses. FFR-guided revascularization has been shown to improve outcomes compared with angiography-guided intervention. Non-invasive FFR derived from CCTA (FFR-CT) represents a promising advancement, allowing functional assessment without invasive instrumentation, though its role in acute settings is still being defined. Instantaneous wave-free ratio (iFR) is a vasodilator-free alternative to FFR that has been validated in large trials and endorsed by guidelines. These physiological assessments are particularly relevant in NSTE-ACS patients with normal ECG, where angiographically intermediate lesions may or may not be functionally significant.

### 6.2 Invasive coronary angiography: indications and findings

Invasive coronary angiography (ICA) remains the definitive diagnostic and therapeutic tool for patients with suspected NSTE-ACS and high-risk features or inconclusive noninvasive assessment. According to the 2023 ESC ACS guidelines, indications for an early invasive strategy (<24 h) include: confirmed NSTEMI (rise/fall of hs-cTn with ≥1 value above the 99th percentile), dynamic ECG changes, GRACE score >140, and temporary rise/rise-fall of troponin. For patients with normal ECG specifically, the decision should be guided by dynamic troponin changes, recurrent symptoms, and high-risk noninvasive test results (3, 16, 25).

The angiographic findings in this population are heterogeneous. While many patients have single- or multivessel atherosclerotic lesions of varying severity, some show normal or near-normal coronary arteries. The latter finding should prompt evaluation of alternative mechanisms, including coronary vasospasm (confirmed by provocation testing), microvascular dysfunction, or spontaneous coronary artery dissection (SCAD) (5, 18). Intravascular imaging (IVUS/OCT) should be employed when angiography is ambiguous or when non-obstructive pathology is suspected. The 2023 ESC guidelines specifically address MINOCA (myocardial infarction with non-obstructive coronary arteries), which represents a significant proportion of normal-ECG NSTE-ACS cases, recommending CMR and intravascular imaging for comprehensive evaluation. FFR or iFR should be used to assess the functional significance of intermediate lesions. For patients with confirmed vasospasm or microvascular angina, the treatment focus should shift from reperfusion to medical therapy with calcium channel blockers, nitrates, and lifestyle modification (5, 38).

## 7 Integrated diagnostic algorithm for suspected NSTE-ACS with normal ECG

**Figure 1:**
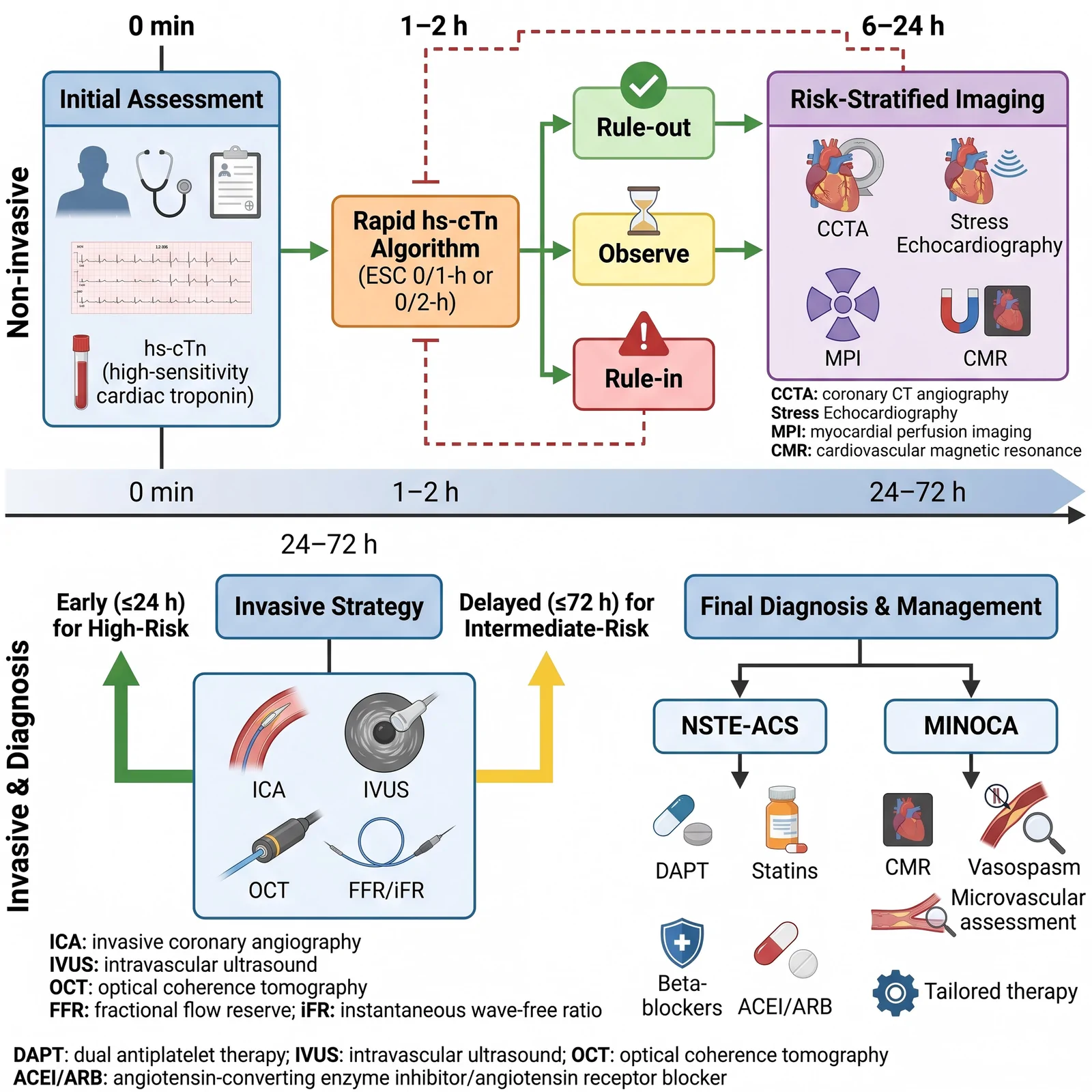
Integrated diagnostic algorithm for suspected NSTE-ACS with normal initial ECG. The algorithm begins with initial triage (12-lead ECG, physical examination, and baseline hs-cTn). ST-elevation prompts immediate STEMI pathway and primary PCI. In patients without ST-elevation, a rapid hs-cTn algorithm (ESC 0/1-h or 0/2-h) triages patients into rule-out, observe, or rule-in pathways. Rule-out patients proceed to anatomical evaluation with coronary CT angiography (CCTA); those with obstructive coronary artery disease (CAD, ≥50% stenosis) undergo functional assessment or invasive coronary angiography (ICA), while those without obstructive CAD may require alternative diagnosis or discharge. Intermediate-risk patients with recurrent symptoms undergo stress echocardiography (with global longitudinal strain, GLS) or myocardial perfusion imaging (MPI), and cardiac magnetic resonance (CMR) is reserved for differential diagnosis. Rule-in patients undergo risk stratification; high-risk patients (GRACE >140, dynamic troponin rise, recurrent ischemia) receive an early invasive strategy (≤24 h), while intermediate-risk patients may undergo a delayed invasive strategy (≤72 h). During ICA, intravascular ultrasound (IVUS)/optical coherence tomography (OCT) characterizes plaque morphology and fractional flow reserve (FFR)/instantaneous wave-free ratio (iFR) assesses functional significance of intermediate lesions. Non-obstructive coronaries prompt a MINOCA work-up (CMR, vasospasm testing, microvascular assessment). Final management includes guideline-directed medical therapy (DAPT, anticoagulation, statins, beta-blockers, ACEI/ARB) for confirmed NSTE-ACS, or tailored therapy for MINOCA/vasospasm/microvascular angina.

**Figure 2:**
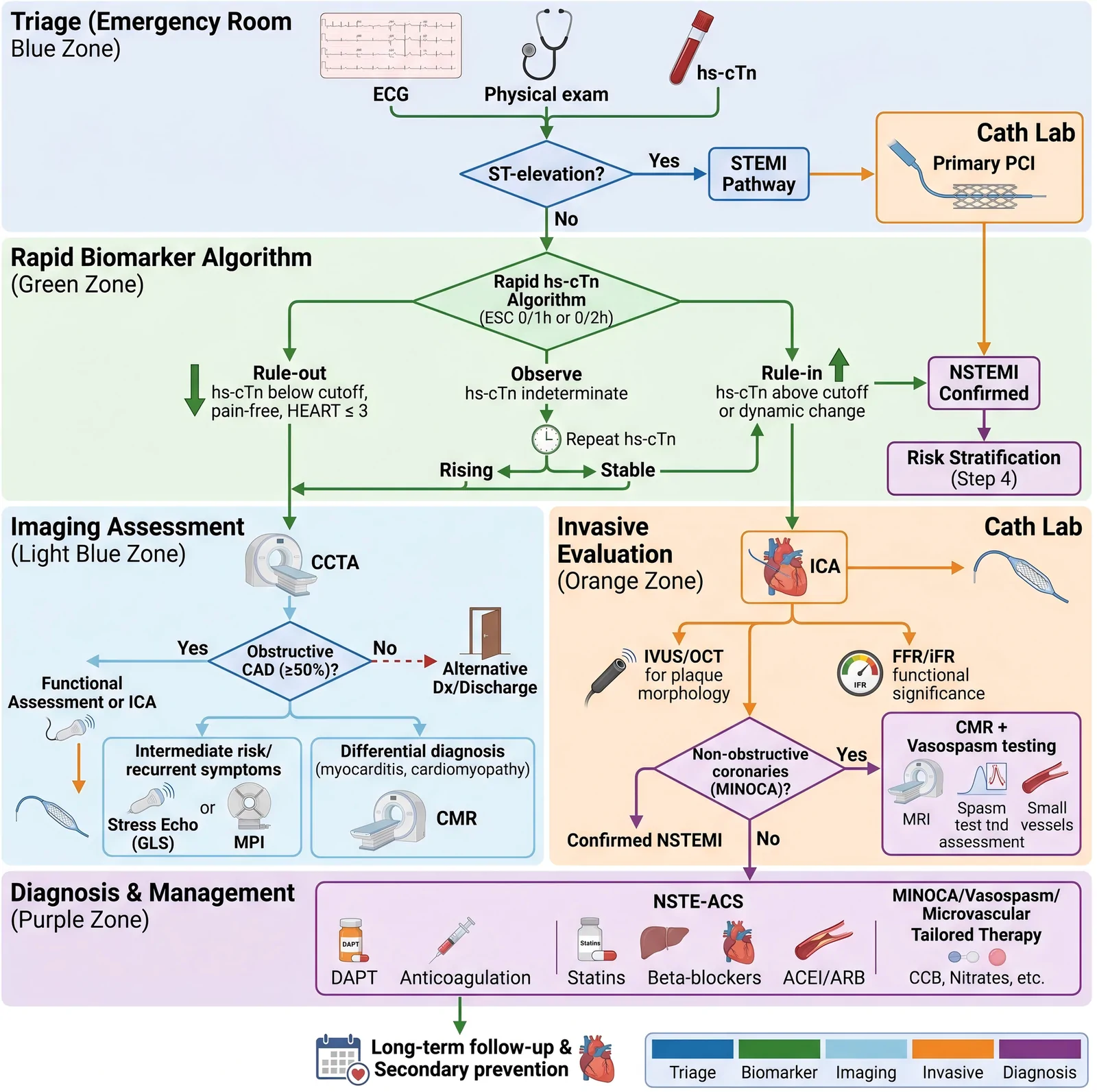
Summary diagnostic pathway for patients with suspected NSTE-ACS and normal initial ECG, illustrating the temporal sequence from emergency department (ED) presentation through triage, rapid hs-cTn algorithm, risk-stratified imaging, and invasive strategy to final diagnosis and management. Upper row (non-invasive): initial assessment at 0 min (12-lead ECG, history, physical examination, and baseline hs-cTn); rapid hs-cTn algorithm at 1–2 h (ESC 0/1-h or 0/2-h protocol) yielding rule-out, observe, or rule-in branches; and risk-stratified imaging at 6–24 h with coronary CT angiography (CCTA), stress echocardiography, myocardial perfusion imaging (MPI), or cardiac magnetic resonance (CMR) selected according to risk profile and clinical presentation. Lower row (invasive and diagnosis): invasive strategy at 24–72 h with invasive coronary angiography (ICA) supplemented by intravascular ultrasound (IVUS)/optical coherence tomography (OCT) and fractional flow reserve (FFR)/instantaneous wave-free ratio (iFR), with early intervention (≤24 h) for high-risk patients and delayed intervention (≤72 h) for intermediate-risk patients. Final diagnosis and management includes NSTE-ACS (guideline-directed medical therapy including dual antiplatelet therapy, statins, beta-blockers, and ACEI/ARB) or MINOCA (tailored therapy after CMR, vasospasm testing, and microvascular assessment). A normal initial ECG must never be used to exclude NSTE-ACS.

A key limitation of the previous version of this review was the separate, non-integrated discussion of ECG, biomarkers, imaging, and risk scores. To address this, we present an integrated diagnostic algorithm (Figure 1) and a summary diagnostic pathway illustrating the complete temporal sequence from emergency department triage through final diagnosis and management (Figure 2) that synthesizes current ESC and ACC/AHA guideline recommendations (48–51) with the specific evidence for the normal-ECG subgroup.

The proposed algorithm proceeds through the following sequential steps:

Step 1: Initial Assessment (Triage)

All patients presenting with suspected ACS undergo immediate 12-lead ECG, physical examination, and initial hs-cTn measurement. If the ECG shows ST-elevation, the patient is managed per STEMI protocols. If the ECG is normal or non-diagnostic but clinical suspicion for NSTE-ACS remains, proceed to Step 2.

Step 2: Rapid hs-cTn Algorithm

Apply the ESC 0/1-h or 0/2-h hs-cTn algorithm (9). Based on the result: (a) Rule-out: if hs-cTn is below the rule-out cutoff and the patient is pain-free with low clinical risk (HEART score ≤ 3), consider CCTA (Step 3a) or discharge with outpatient follow-up; (b) Observe: if hs-cTn is in the observation zone, repeat hs-cTn at the designated interval and continue monitoring; (c) Rule-in: if hs-cTn is above the rule-in cutoff or shows a dynamic rise/fall pattern, the diagnosis of NSTEMI is confirmed—proceed to risk stratification (Step 4) and consider early invasive strategy.

Step 3: Noninvasive Imaging (Risk-Stratified)

(3a) Low-to-intermediate risk (HEART ≤ 6, negative/indeterminate hs-cTn): CCTA is the preferred initial modality to exclude obstructive CAD. If CCTA shows obstructive disease (≥50% stenosis), proceed to functional assessment (stress imaging or FFR-CT) or ICA. If CCTA is negative, consider alternative diagnoses or outpatient follow-up. (3b) Intermediate risk with recurrent symptoms: stress echocardiography (with GLS assessment) or MPI to detect inducible ischemia. If positive, proceed to ICA. (3c) Inconclusive initial workup or suspected ACS mimic: CMR for tissue characterization and differential diagnosis (myocarditis, Takotsubo, microinfarction).

Step 4: Risk Stratification and Invasive Strategy Decision

For confirmed NSTEMI patients: apply GRACE score and assess high-risk criteria. High-risk patients (GRACE >140, dynamic troponin, recurrent ischemia, hemodynamic instability) warrant early invasive strategy (ICA within 24 h). Intermediate-risk patients may undergo delayed invasive strategy (within 72 h). During ICA, employ IVUS/OCT for ambiguous lesions and FFR/iFR for intermediate stenoses. If coronary arteries are normal or non-obstructive (MINOCA), perform CMR and evaluate for coronary vasospasm (provocation testing), microvascular dysfunction, or SCAD.

Step 5: Diagnosis Confirmation and Management

Based on the integrated findings, initiate guideline-directed medical therapy (DAPT, anticoagulation, statins, beta-blockers, ACEI/ARB) for confirmed NSTE-ACS. For MINOCA or microvascular angina, tailor therapy to the underlying mechanism. Ensure appropriate follow-up and secondary prevention.

## 8 Treatment strategies, prognosis, and management implications

### 8.1 Pharmacotherapy and timing of invasive strategy

Once NSTE-ACS is diagnosed, guideline-directed medical therapy should be initiated immediately regardless of ECG findings, as recommended by both the 2023 ESC ACS guidelines and the 2014 ACC/AHA NSTE-ACS guideline. Standard pharmacotherapy includes dual antiplatelet therapy (DAPT: aspirin plus a P2Y12 inhibitor—preferably ticagrelor or prasugrel), anticoagulation (unfractionated heparin, enoxaparin, or fondaparinux), high-intensity statins, beta-blockers, and ACE inhibitors or ARBs, particularly in patients with diabetes, hypertension, or left ventricular dysfunction. Evidence confirms that even patients with normal ECG but elevated troponin have significant myocardial damage requiring active treatment (1, 2). However, no randomized controlled trial has specifically evaluated the benefit of guideline-directed medical therapy in the normal-ECG NSTE-ACS subgroup; current recommendations are necessarily extrapolated from broader ACS trials that did not stratify by ECG findings at presentation.

The timing of invasive management in the normal-ECG subgroup merits careful consideration. The 2023 ESC guidelines recommend an early invasive strategy (<24 h) for high-risk NSTE-ACS patients, including those with confirmed NSTEMI, dynamic biomarker changes, or GRACE score >140. However, direct evidence specifically addressing patients with normal ECG is limited, as most randomized trials of early invasive strategies (e.g., FRISC-II, RITA-3, ICTUS) did not specifically stratify by ECG findings (54). This is a notable gap in the evidence. Decision-making should therefore be individualized, integrating risk stratification tools, dynamic troponin changes, and symptom stability (3, 16). For patients with confirmed vasospasm or microvascular angina, the treatment paradigm shifts to calcium channel blockers, nitrates, and lifestyle modification (5, 38).

### 8.2 Short-term and long-term prognosis

When NSTE-ACS patients present with normal ECG but elevated troponin, they face significant cardiovascular risk. Compared with non-ACS chest pain patients, they have higher rates of MACE (death, myocardial infarction, and revascularization) at 30 days and 1 year, although the risk may be somewhat lower than in patients with overt ECG changes (1–3). A critical limitation of the available prognostic data is that most estimates derive from single-center observational studies with relatively small sample sizes and limited adjustment for confounders, which may affect the precision and generalizability of these risk estimates. The available prognostic data, it is worth noting, predominantly derive from single-center observational studies with relatively small sample sizes, limiting the precision of risk estimates. Large, multicenter registries specifically addressing the normal-ECG NSTE-ACS subgroup are needed.

What predicts poor outcomes? Several factors have been linked to adverse outcomes: advanced age, elevated peak troponin, and multivessel coronary disease. Left ventricular systolic dysfunction and non-adherence to guideline-directed therapy also contribute (3, 15). Of note, microvascular dysfunction has been identified as an independent predictor of poor prognosis, emphasizing the need for comprehensive management addressing both epicardial and microvascular disease (39, 40). The study by Kraen et al. (40), published in Heart, demonstrated the prognostic importance of exercise ECG in patients with suspected microvascular disease, providing evidence that functional testing has value beyond simple ischemia detection. These findings collectively demonstrate that a normal ECG in NSTE-ACS does not equate to a benign clinical course, reinforcing the need for vigilant risk assessment and longitudinal follow-up.

### 8.3 Implications for clinical practice and guideline integration

Several take-home messages emerge for clinical practice. The 2023 ESC ACS guidelines and the 2021 ACC/AHA Chest Pain Evaluation guidelines both emphasize the central role of hs-cTn and structured risk stratification. Key recommendations relevant to the normal-ECG subgroup include: (1) use of rapid hs-cTn algorithms (0/1-h or 0/2-h) for all patients with suspected ACS regardless of ECG findings (ESC Class I, Level A); (2) CCTA for low-risk patients (HEART ≤ 3) with negative hs-cTn to exclude obstructive CAD (ACC/AHA Class I); (3) early invasive strategy for confirmed NSTEMI with high-risk features (ESC Class I, Level A); (4) comprehensive MINOCA evaluation with CMR and intravascular imaging when coronary arteries are non-obstructive (ESC Class IIa).

In our view, future guideline updates should specifically address the normal-ECG subgroup, providing explicit recommendations on the role of advanced imaging, the duration of ECG monitoring, and the optimal threshold for invasive evaluation in this heterogeneous population. Greater attention to non-obstructive CAD subtypes (microvascular angina, vasospasm, SCAD) is also needed, as these represent a substantial proportion of normal-ECG NSTE-ACS cases and have distinct management requirements.

## 9 Future perspectives

The diagnosis and management of NSTE-ACS with normal ECG continue to evolve, with several new technologies that could change practice.

### 9.1 Artificial intelligence-assisted ECG interpretation

Artificial intelligence (AI) and deep learning models applied to ECG signals have shown considerable ability to detect subtle ischemic patterns imperceptible to the human eye. The study by Hori et al. (14) showed that a convolutional neural network (CNN)-enhanced ECG algorithm could detect ACS, including NSTE-ACS subtypes, with improved sensitivity compared to standard interpretation. AI-ECG models trained on large datasets can identify hidden signatures of ischemia, repolarization abnormalities, and high-risk coronary anatomy. While early results are encouraging, clinical translation remains limited. These models, though, require prospective multicenter validation, and concerns about algorithmic bias, generalizability across different populations, and regulatory approval must be addressed before clinical implementation.

### 9.2 Machine learning prediction models

Machine learning (ML) approaches integrating multidimensional clinical, biomarker, and imaging data have been explored as potential tools that may complement traditional scoring systems for risk prediction, although prospective validation is lacking. The study by Tsivanyuk et al. (33) demonstrated the efficiency of ML in predicting obstructive CAD in NSTE-ACS patients within the first hours of admission. However, robust external validation across diverse clinical settings has not yet been demonstrated. Future ML models could integrate real-time hs-cTn kinetics, ECG features, clinical variables, and imaging findings to generate individualized risk scores, enabling precision-based triage decisions. Challenges include the need for large, high-quality training datasets, model interpretability (the “black box” problem), and integration into clinical workflows.

### 9.3 Multimodal diagnostic algorithms

It is worth emphasizing that the integration of biomarker, imaging, and clinical data into unified diagnostic algorithms represents the future direction for optimizing NSTE-ACS diagnosis in normal-ECG patients. Point-of-care multimodal platforms that combine rapid hs-cTn testing with AI-enhanced ECG analysis and FFR-CT could streamline the diagnostic pathway, reducing time to definitive diagnosis and treatment. The ongoing TRACTION trial (35) and COURSE trial (10) will provide important evidence for incorporating CCTA into the acute care pathway.

### 9.4 Wearable ECG technologies

Increasingly, wearable devices capable of continuous ECG monitoring could fundamentally change how we detect transient ischemic episodes that would otherwise be missed. Smartwatch-based single-lead ECG, wearable patch monitors, and implantable loop recorders enable prolonged rhythm and ST-segment monitoring in ambulatory patients. The wearable infrared sensor described by Sengupta et al. (41) for detecting NSTE-ACS represents an innovative approach, though it remains investigational. These technologies are particularly promising for patients with recurrent chest pain and normal initial ECG, potentially capturing ischemic events during daily activities. But issues of signal quality, data overload, false alarms, and reimbursement need to be resolved.

### 9.5 Precision medicine approaches

Precision medicine aims to tailor diagnostic and therapeutic strategies to individual patient characteristics, including genetic predisposition, biomarker profiles, and imaging phenotypes. In the context of NSTE-ACS with normal ECG, precision approaches could identify patients most likely to benefit from specific interventions (e.g., FFR-CT for intermediate lesions, CMR for MINOCA evaluation, targeted therapy for vasospastic angina). Genomic and proteomic biomarker research may identify novel risk markers specific to the normal-ECG phenotype. The integration of multi-omics data with clinical and imaging information through AI-driven analytics could ultimately enable truly personalized ACS management.

### 9.6 Identified knowledge gaps and research priorities

This review identifies several critical knowledge gaps that should guide future research: (1) large, multicenter prospective registries specifically enrolling NSTE-ACS patients with normal ECG are needed to provide robust prevalence, outcome, and prognostic data; (2) validation of rapid hs-cTn algorithms specifically in the normal-ECG subgroup across diverse populations and assay platforms; (3) randomized trials comparing CCTA-first vs. standard pathways in normal-ECG patients; (4) prospective studies of AI-enhanced ECG and ML prediction models in multicenter settings; (5) standardized protocols for MINOCA evaluation incorporating CMR, intravascular imaging, and vasoreactivity testing; (6) studies of wearable monitoring technologies for detecting transient ischemia in ambulatory patients with normal baseline ECG. Addressing these gaps will be essential for developing evidence-based guidelines specific to this challenging patient subgroup.

## 10 Conclusion

The identification and management of NSTE-ACS in patients presenting with normal ECG remains a significant and evolving challenge in contemporary cardiology. This review has synthesized the current evidence, critically evaluated the strengths and limitations of available diagnostic tools, and presented an integrated diagnostic algorithm that aligns with current ESC and ACC/AHA guideline recommendations.

Several key messages emerge from this review. First and foremost, a normal ECG must never be used to exclude NSTE-ACS. Clinical suspicion, guided by symptom assessment and serial hs-cTn measurement, remains the cornerstone of diagnosis. Rapid hs-cTn algorithms (0/1-h or 0/2-h) provide high diagnostic accuracy even in the absence of ECG changes and should be universally applied. Beyond biomarkers, risk-stratified imaging plays a critical complementary role—CCTA for anatomical assessment in low-to-intermediate-risk patients, stress echocardiography and MPI for functional ischemia detection, CMR for tissue characterization and differential diagnosis, and intravascular imaging (IVUS/OCT) with physiological assessment (FFR/iFR) during invasive evaluation. An integrated, multimodal diagnostic pathway—as presented in this review—optimizes both diagnostic accuracy and timeliness. Finally, it bears repeating that despite the absence of ECG changes, confirmed NSTE-ACS patients carry substantial cardiovascular risk, necessitating guideline-directed medical therapy and individualized invasive strategy decisions.

Looking forward, artificial intelligence-assisted ECG interpretation, machine learning prediction models, wearable monitoring technologies, and precision medicine approaches hold significant promise for improving diagnostic sensitivity and personalizing management. However, these technologies require rigorous prospective validation before clinical implementation. Addressing the identified knowledge gaps through well-designed multicenter studies will be essential for developing evidence-based guidelines specific to this complex and clinically significant patient subgroup. Ultimately, the paradigm shift from relying on ECG alone toward a comprehensive, multimodal, and individualized approach will improve diagnostic accuracy, reduce missed diagnoses, and optimize outcomes for patients with NSTE-ACS and normal initial ECG.
